# CHATGPT, WHAT IS A DEDUCTIBLE? DIGITAL ASSISTANTS AS AN INFORMATION SOURCE FOR MEDICARE QUERIES

**DOI:** 10.1093/geroni/igad104.3220

**Published:** 2023-12-21

**Authors:** Emily Langston, Neil Charness, Walter Boot

**Affiliations:** Florida State University, Tallahassee, Florida, United States; Florida State University, Tallahassee, Florida, United States; Florida State University, Tallahassee, Florida, United States

## Abstract

Since being first introduced to the public in late 2022, chatbots that are based on large language models (LLMs) have attracted a great deal of attention. However, despite their popularity, these chatbots have been shown to give inaccurate information in response to user queries. Several polls conducted in 2022 suggest that the public is generally skeptical about the use of Artificial Intelligence (AI), particularly in the context of healthcare, and older adults are the most distrustful age group, despite the fact that they are the least likely to have interacted with an LLM such as ChatGPT. Meta-analysis on trust in AI has shown that users are most influenced by the accuracy and reliability of the AI trustee. In our study, we assessed the accuracy of speaker-based assistants, Alexa and Google Assistant, as well as two LLMs, Bard and ChatGPT4 on Medicare terminology and knowledge and compared their accuracy to that of a large representative sample of Medicare beneficiaries. Google Assistant was found to perform significantly worse than beneficiaries on both terminology and knowledge questions. Alexa was found to perform significantly worse than beneficiaries on terminology questions. Conversely, both Bard and ChatGPT4 were found to perform significantly better than beneficiaries on both terminology and knowledge questions. We conclude that Medicare beneficiaries should not rely on Google Assistant for terminology help or general knowledge queries, nor should they rely on Alexa for terminology help. ChatGPT4 and Bard are potentially valuable resources for beneficiaries with terminology-based and general knowledge queries.

